# Assessing the *In Vitro* Digestion of Lactoferrin-Curcumin Nanoparticles Using the Realistic Gastric Model

**DOI:** 10.3390/nano13152237

**Published:** 2023-08-02

**Authors:** Daniel A. Madalena, João F. Araújo, Óscar L. Ramos, António A. Vicente, Ana C. Pinheiro

**Affiliations:** 1 CEB—Centre of Biological Engineering, University of Minho, 4710-057 Braga, Portugal; 2 LABBELS—Associate Laboratory, 4710-057 Braga/Guimarães, Portugal; 3 CBQF—Centro de Biotecnologia e Química Fina, Escola Superior de Biotecnologia, Universidade Católica Portuguesa/Porto, 4202-401 Porto, Portugal

**Keywords:** *in vitro* digestion, dynamic gastric digestion, nanodelivery system, nanostructures, bioaccessibility

## Abstract

Nanosized delivery systems have been the subject of research and discussion in the scientific community due to their unique properties and functionality. However, studies reporting the behaviour of nanodelivery systems under dynamic *in vitro* digestion conditions are still very scarce. To address this gap, this study aims to assess the dynamic *in vitro* gastric digestion of lactoferrin/curcumin nanoparticles in the realistic gastric model (RGM). For this purpose, the INFOGEST standard semi-dynamic digestion protocol was used. The nanosystems were characterized in terms of hydrodynamic size, size distribution, polydispersity index (PdI), and zeta potential using dynamic light scattering (DLS), before and during the digestion process. Confocal laser scanning microscopy (CLSM) was also used to examine particle aggregation. In addition, the release of curcumin was evaluated spectroscopically and the intrinsic fluorescence of lactoferrin was measured throughout the digestion process. The protein hydrolysis was also determined by UV-VIS-SWNIR spectroscopy to estimate, in real-time, the presence of free NH2 groups during gastric digestion. It was possible to observe that lactoferrin/curcumin nanoparticles were destabilized during the dynamic digestion process. It was also possible to conclude that low sample volumes can pose a major challenge in the application of dynamic *in vitro* digestion models.

## 1. Introduction

Nanosized delivery systems have been the subject of research and discussion in the scientific community due to their unique properties and functionality, despite the regulatory aspects limiting their application to food products [1]. These systems provide protection to bioactive compounds (e.g., curcumin) that otherwise could be exposed to detrimental environmental conditions (e.g., light, oxygen, and exposure to digestion conditions, among others). Materials at the nanometre scale present, e.g., a higher surface area to volume ratio [2]. This means that, due to their unique properties, nanosized delivery systems can be added to food to produce functional foods that present additional benefits beyond their nutritional profile. Generally recognized as safe (GRAS) materials need to be used since these systems are intended to be applied to food products that will be consumed and digested [2,3]. For this reason, bio-based delivery nanosystems, which are considered GRAS materials, have been used as potential encapsulating agents to protect and control the release of bioactive compounds for food applications [2]. Thus, bio-based delivery nanosystems (e.g., lactoferrin nanoparticles) present a huge potential to be explored in food products; however, studies showing its behaviour under digestion conditions are of utmost importance [4].

Lactoferrin (Lf), a protein present in milk, exhibits by itself several interesting properties, including antibacterial, antifungal, antiparasitic, antiviral, anticarcinogenic, and anti-inflammatory properties [3]. Despite the mentioned properties, this protein can also be used to improve the water solubility of lipophilic bioactive compounds such as curcumin. Curcumin, in turn, is a polyphenolic compound that has received great interest in the scientific community due to its well-documented anti-inflammatory, antimicrobial, anticarcinogenic, and antioxidant properties [5,6]. Regarding Lf’s digestion, the studies reported in the literature seem to be inconsistent, as some research and review articles report that Lf is completely digested in the stomach [3,7,8], while other studies report that Lf presents some degree of resistance to the enzymatic digestion in the stomach, being completely digested in the small intestine [9]. Furthermore, there is still a lack of studies regarding Lf’s digestibility under dynamic *in vitro* digestion conditions. This study evaluates the performance of previously developed Lf nanoparticles loaded with curcumin in a realistic gastric model (RGM), a dynamic realistic gastric model that mimics the physio-anatomical characteristics of the human stomach, using the semi-dynamic standard *in vitro* digestion protocol developed by Mulet-Cabero et al. [10]. Particle characterization before and during the *in vitro* digestion process was performed by dynamic light scattering (DLS) (i.e., for particles’ hydrodynamic size, size distribution, polydispersity index (PdI), and zeta potential) and confocal laser scanning microscopy (CLSM) (i.e., to assess particle aggregation). Furthermore, the release of curcumin was also analysed spectroscopically, and the intrinsic fluorescence of Lf was measured during the digestion process to evaluate the exposure of tryptophan residues. Moreover, protein hydrolysis was measured using ultraviolet-visible-shortwave near infrared (UV-VIS-SWNIR) spectroscopy to estimate the free NH_2_ groups during the *in vitro* gastric digestion process.

## 2. Materials and Methods

### 2.1. Materials

Purified bovine Lf, composed of at least 96% protein (value informed by the manufacturer), was purchased from DMV International (Coral Gables, FL, USA). The reagents KCl, KH_2_PO_4_, NaHCO_3_, NaCl, MgCl_2_(H_2_O)_6_, (NH_4_)_2_CO_3_, FITC, pepsin from porcine gastric mucosa (1264 U.mg^−1^), and curcumin were purchased from Merck (Darmstadt, Germany).

### 2.2. Lf–Curcumin Nanoparticles Preparation

Lf nanoparticles loaded with curcumin were prepared following the protocol reported elsewhere [5]. Briefly, 100 mL of 0.2% (*w*/*v*) Lf solution was prepared at room temperature and stirred for 1 h. The pH of the Lf solution was subsequently adjusted to 7.0 using 0.1 mol·L^−1^ of NaOH and further left overnight at 4 °C to promote a complete protein hydration. The samples were then heated at 75 °C for 10 min in a water bath. After the thermal treatment, a stock solution of curcumin (i.e., 1000 μg·mL^−1^ dissolved in pure ethanol) was added to reach a final concentration of 80 μg·mL^−1^, which was previously optimized in the work of Araújo et al. [5]. The samples were then cooled at room temperature for at least 30 min prior to digestion.

To determine the association efficiency (*AE*) of Lf–curcumin nanoparticles, the samples were centrifuged at 12,000× *g* for 20 min at 4 °C to precipitate the undissolved curcumin located in the pellet. The pellet was further resuspended with pure ethanol and analysed spectroscopically at 425 nm according to the works of Bourbon et al. and Araújo et al. [5,11]. The amount of free curcumin was calculated using the calibration equation y=122.63x+0.0184, r^2^ = 0.9994. The *AE* was further calculated using Equation (1):

(1) $$AE\left(\%\right)=\frac{{Total}_{curcumin}-{Free}_{curcumin}}{{Total}_{curcumin}}*100$$

### 2.3. The In Vitro RGM

**Figure 1:**
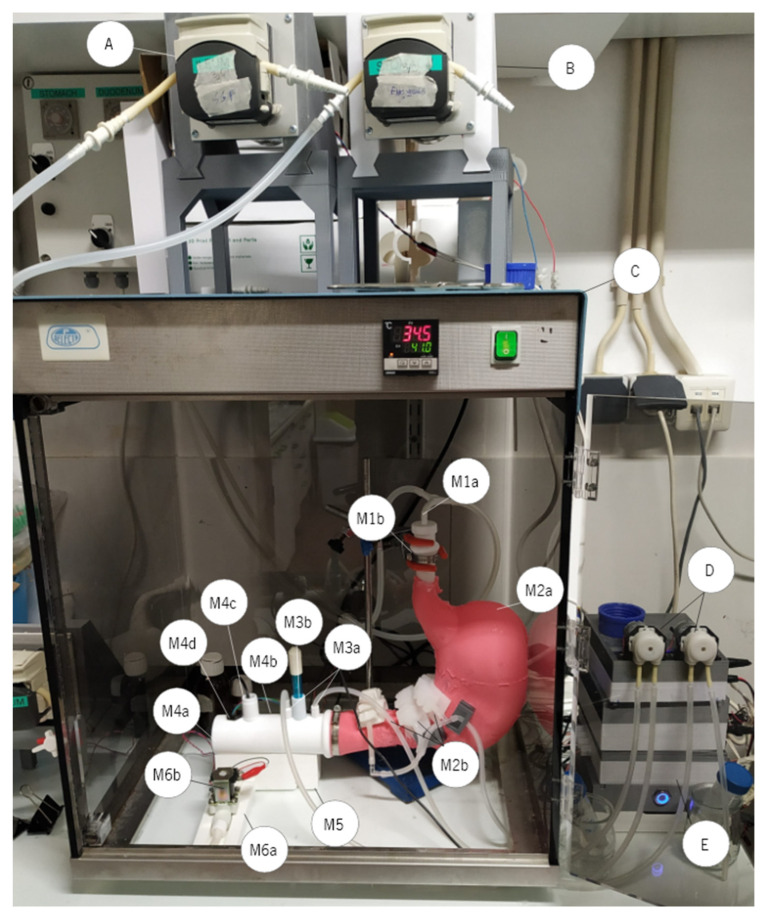
The *in vitro* realistic gastric model, where: M1a—SGF and enzymes input; M1b—module 1 connection with module 2; M2a—module 2; M2b—peristaltic bands; M3a—acid/base input; M3b—pH electrode support; M4a—module 4; M4b—pylorus; M4c—fibre optics support; M4d—level sensor; M5—module 5; M6a—module 6; M6b—electrovalve; A—SGF peristaltic pump; B—enzymes peristaltic pump; C—standard laboratory oven; D—acid/base peristaltic pumps; E—control unit.

The primary goal in developing the RGM (Figure 1) was to develop a dynamic *in vitro* gastric model that could be easily replicated, with the potential to establish it as a standard dynamic *in vitro* gastric model. It is comprised of six modules, with module 1 designed to accommodate the input of the sample, gastric fluid (SGF), and enzymes. Module 2 represents the J-shaped silicone gastric compartment (i.e., similar to the human stomach), while module 3 serves as the pH electrode support. Module 4 provides support for the pylorus and level sensor, module 5 holds modules 3 and 4 together, and module 6 supports the electrovalve. Furthermore, to accurately replicate the peristaltic contractions within the gastric compartment, three peristaltic bands (PBs) were strategically placed in physiologically significant locations based on the research of Ferrua and Singh [12]. These PBs, labelled PB1, PB2, and PB3, correspond to contractions of 30%, 40%, and 80% intensity, respectively. This intensity gradient is designed to mimic the increasing contraction amplitude as the contractions approach the pylorus, simulating antral wave contractions that occur in the human stomach. To maintain a consistent digestion temperature, a standard laboratory oven is employed, ensuring that the temperature remains at 37 °C throughout the digestion process.

### 2.4. In Vitro Digestion Protocol

The INFOGEST semi-dynamic *in vitro* digestion protocol [10] was used to assess the gastric digestion of Lf nanoparticles loaded with curcumin. The *in vitro* digestion conditions were calculated according to the samples’ nutritional composition. However, since the analysed samples contained only 0.2% protein, the residence time in the stomach would be too short. As such, it was considered that this structure would be applied to a food product, e.g., a yoghurt containing 2.7 g of protein, 1.8 g of lipids, and 3.4 g of carbohydrates in 100 g of yoghurt. This nutritional information was further used to calculate the digestion parameters which are detailed in Table 1. Subsequently, 100 mL of Lf–curcumin nanoparticles were mixed with simulated salivary fluid (SSF), 0.3 mol·L^−1^ CaCl_2_, and water. Salivary amylase was not added, since the sample did not contain starch (the corresponding volume of distilled water was used instead), and the oral phase started with a duration of 2 min. The contents were transferred into the RGM which contained a basal volume of 7.21 mL of SGF and 0.3 mol·L^−1^ CaCl_2_. Both SGF and enzymes were gradually pumped at a flow rate of 3.24 and 0.39 mL·min^−1^, respectively, with a gastric digestion duration of 26:43 min. Pepsin activity (i.e., 1264 U/mL) was determined using the protocol described by Mulet-Cabero et al. [10].

The release of curcumin was determined using a similar procedure to the one described in topic 2.2 This way, samples from each stomach emptying were centrifuged at 12,000× *g* for 20 min at 4 °C to precipitate the undissolved curcumin located in the pellet. The pellet was further resuspended with pure ethanol and analysed spectroscopically at 425 nm according to the works of Bourbon et al. and Araújo et al. [5,11]. The amount of free curcumin was calculated using the calibration equation y=122.63x+0.0184, r^2^ = 0.9994. The absorbance value of each sample was corrected using the dilution factor for each stomach emptying. The corrected absorbance values were then compared with the initial curcumin absorbance (Equation (2)).

(2) $${Curcumin}_{released}\left(\%\right)=\frac{{Abs}_{SE}*DF}{{Total}_{curcumin}}*100$$

where, *curcumin_released_* corresponds to the amount of curcumin released in a stomach emptying sample, *Abs_SE_* corresponds to the absorbance of free curcumin in the stomach emptying sample, *DF* corresponds to the dilution factor, and *Total_curcumin_* corresponds to the initial curcumin’s absorbance.

### 2.5. Dynamic Light Scattering

The Lf–curcumin nanoparticles were characterized in terms of their particle hydrodynamic size, particle distribution, PdI, and zeta potential. For this purpose, DLS (Zetasizer Nano ZS, Malvern Instruments, Malvern, United Kingdom) equipped with a He-Ne laser with a wavelength of 633 nm was used. This technique was used before and during the digestion process to monitor changes in the Lf–curcumin nanoparticles’ stability during the digestion process. The DLS results were further validated by analysing the autocorrelation function, since it can be used to assess the quality of the DLS results [13].

### 2.6. Lf Intrinsic Fluorescence

To assess the exposure of tryptophan residues in Lf nanoparticles, the protein intrinsic fluorescence was used. The measurements were conducted on all stomach emptying samples using a microplate reader (Biotech Synergy HT, Biotek, Winooski, VT, USA) with a fluorescence-appropriate microplate. A calibration with pepsin (${FI}_{pepsin}=362136.0*P+5331.4$, where *FI_pepsin_* corresponds to the fluorescence intensity of pepsin and P corresponds to the pepsin concentration) was used to eliminate the influence of pepsin intrinsic fluorescence during the digestion process. The fluorescence analysis was conducted using an excitation wavelength of 280 nm and an emission wavelength of 350 nm [14].

### 2.7. Confocal Scanning Laser Microscopy

Microscopy images were taken using CSLM (Olympus BX61, Model FluoView 1000, Shinjuku City, Japan) to characterize the samples prior to digestion, as well as to assess the aggregation of Lf–curcumin nanoparticles during the *in vitro* digestion process, using a 60× oil-immersed objective lens. The samples of native Lf, denatured Lf, and Lf–curcumin nanoparticles, as well as samples from stomach emptying 4 and 8 (i.e., digestion times of 12.68 and 25.38 min, respectively) were stained according to Liang et al. [15] Briefly, a volume of 5 µL of FITC (10 mg·mL^−1^ in dimethyl sulfoxide) was added to 200 µL of sample. The stained samples were then vortexed for 5 min and 7 µL were transferred to the microscope slide. The samples were then analysed using a green laser (laser with the reference laser488 BA: 505–540) since the emission and excitation wavelengths of FITC are 488 and 545 nm, respectively. All images were acquired and processed with the software FV10-Ver4.1.1.5 (Olympus).

### 2.8. Real-Time Analyses of Lf Nanoparticles during In Vitro Gastric Digestion

The *in vitro* Lf–curcumin nanoparticles’ digestion was also evaluated via UV-VIS-SWNIR spectroscopy using a fibre-optic (T300-RT-VIS/NIR, Oceanoptics, Orlando, FL, USA) multichannel spectrophotometer (AvaSpec-2048-DT-4, 2048-pixel, 200–1100 nm). A halogen light source (AvaLight-Hal LS-0308016, 360–2500 nm, Avantes, Apeldoorn, The Netherlands) was used since it covers the region of interest of the spectrum (i.e., 360–1100 nm). The spectra were subsequently acquired using AvaSoft 7.5.3 by averaging 30 spectra on each acquisition. The spectra data were further used to estimate the release of amino acids during the digestion process using the random forest regression (RFR) algorithm as a regression model, which was selected in a comparison between several regression models (e.g., multiple linear regression, ridge regression, lasso regression, elastic net regression, Bayesian ridge regression, support vector machine regression and random forest regression). The number of estimators was set to 1000 estimators which produced a correlation coefficient of 0.9988 (the number of estimators was determined by optimizing the RFR model using an experimental design). It is important to mention that it was only possible to acquire spectra data until stomach emptying 3 (i.e., 9:31 min) due to the low volume inside the RGM, which was not sufficient to completely immerse the fibre-optic transmission probe.

### 2.9. Statistical Analysis

All statistical analyses applied to experimental data were performed using Origin (2018, OriginLab Corporation, Northampton, MA, USA), unless indicated otherwise in the text. The statistical significance (at *p* ≤ 0.05) was determined using one-way ANOVA followed by post hoc Tukey’s tests with at least triplicate samples, unless mentioned otherwise in the respective section. 

## 3. Results and Discussion

### 3.1. Lf–Curcumin Nanoparticles’ Characterization

Lf–curcumin nanoparticles were used to assess the performance of the RGM in the digestion of nanodelivery systems. Therefore, a particle characterization was conducted prior to the *in vitro* digestion in terms of particle hydrodynamic size, PdI, and zeta potential, as well as association efficiency. The results are shown in Table 2.

**Figure 2:**
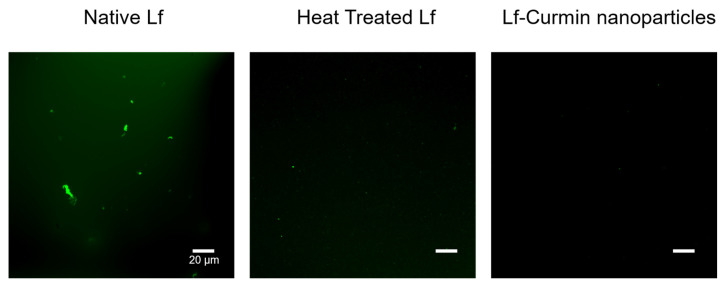
CLSM images of native Lf, heat-treated Lf, and Lf–curcumin nanoparticles. The white bars represent 20 µm.

From the analysis of Table 2, it is possible to conclude that Lf–curcumin nanoparticles were successfully produced with a particle hydrodynamic size of 118.97 ± 12.45 nm. The particles showed a PdI of 0.24 ± 0.10, which indicates a homogeneous system since the nanoparticles’ PdI is <0.3, [16] and a zeta potential of 10.04 ± 2.16, a positive value since Lf is a positively charged protein at pH values below its isoelectric point (i.e., 8.0–9.0) [8]. The Lf–curcumin nanoparticles presented an association efficiency of 82.10 ± 0.04%, which indicates that Lf was able to associate to curcumin with a high efficiency which is in accordance with previous works [5,17,18]. However, despite the similarity in the particles’ hydrodynamic size and PdI of 100.00 ± 5.00 nm and 0.3, respectively, Bollimpelli et al. [17] reported a negative zeta potential value of −19 mV, at pH 7.4 (i.e., below the isoelectric point of Lf), and an association efficiency of 61.30 ± 2.40%. The authors do not explain the negative charge in the particles, but it can perhaps be related to the preparation procedure used by the authors, i.e., a cold sol-oil procedure where the samples are mixed with dimethyl sulfoxide (DMSO) and phosphate-buffered saline (PBS) solution. Since the Lf structure is dependent on the ionic strength of the media, among other parameters [3,8] the use of salts, e.g., PBS, could be responsible for the negative charge observed in the nanoparticles. Furthermore, the Lf–curcumin nanoparticles were also characterized using CSLM to microscopically observe the influence of protein denaturation and the addition of curcumin. The results are depicted in Figure 2.

It is possible to observe in Figure 2 that Lf samples prior to heat treatment present some heterogeneity, since large particle agglomerates with ca. 20 µm of diameter can be identified along with small protein particles. It is also possible to observe that heating the Lf samples at 75 °C promoted the solubilization of large particles and a more homogeneous particle distribution. Furthermore, the addition of curcumin to the Lf samples did not influence their size.

### 3.2. Lf–Curcumin Nanoparticles’ Behaviour during In Vitro Gastric Digestion

**Figure 3:**
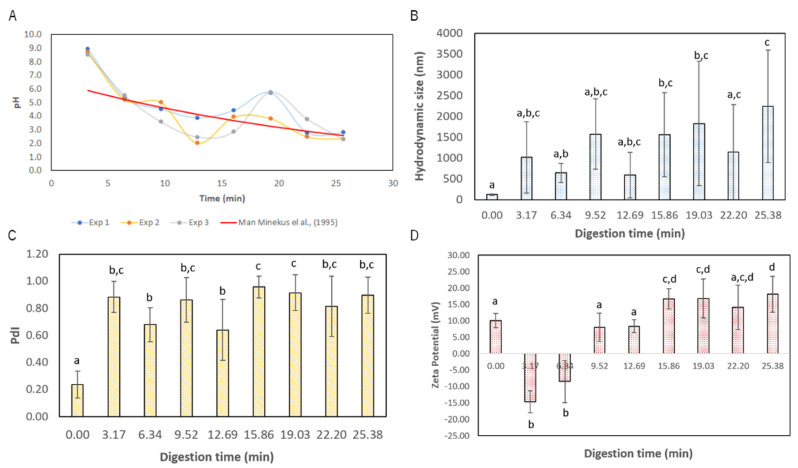
Gastric digestion pH (**A**) and Lf–curcumin nanoparticles’ size (**B**), PdI (**C**), and zeta potential (**D**) during the *in vitro* gastric digestion. Samples with the same letters indicate that there is no significant statistical difference (*p*-value > 0.05).

The Lf–curcumin nanoparticles were submitted to a dynamic *in vitro* gastric digestion protocol in the RGM and their hydrodynamic size, size distribution, PdI, and zeta potential were monitored during this procedure using DLS. The results are depicted in Figure 3.

**Figure 4:**
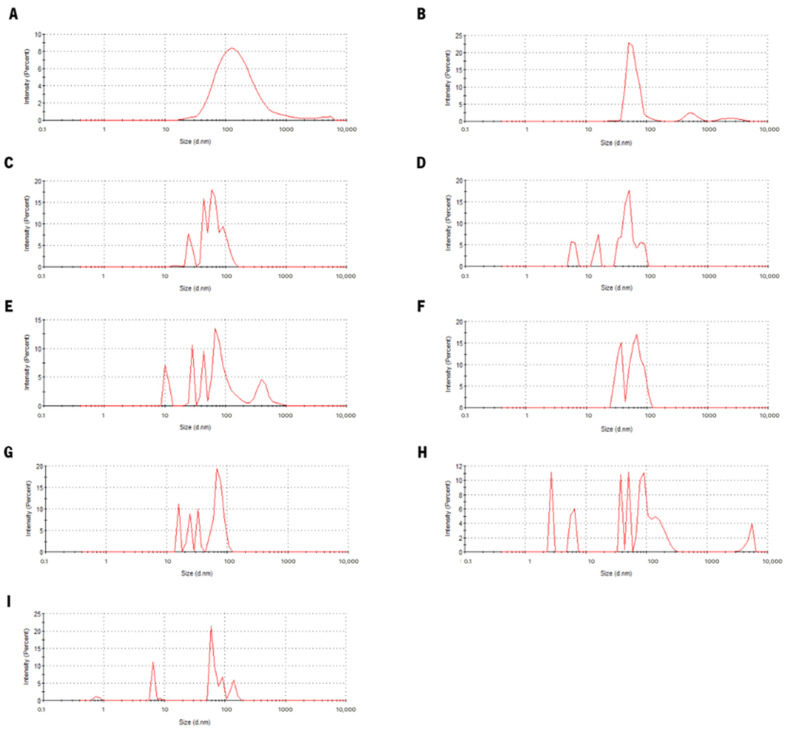
Particle size distribution of Lf–curcumin nanoparticles before (**A**) and after (**B**–**I**) the digestion process, where the letters from B to I correspond to the stomach emptying times from 1 to 8, respectively.

It is possible to observe from the analysis of Figure 3 that the Lf–curcumin nanoparticles were rapidly destabilized, which is indicated by a significant (*p*-value < 0.05) increase in PdI from 0.24 ± 0.10 to 0.88 ± 0.11, when comparing the initial state of the Lf–curcumin nanoparticles with the first stomach emptying (i.e., digestion time of 3.17 min). However, due to the high standard deviation, there is no statistically significant difference between the samples obtained at 3.17, 6.34, 9:31, 12.68, and 22.19 min, despite the increase in the average hydrodynamic particle size from 118.97 ± 12.45 nm to 1012.61 ± 858.61 nm. This indicates that ANOVA with a means comparison using Tukey’s test is not appropriate for assessing the statistical difference throughout the digestion process due to the high standard deviation. Furthermore, the zeta potential of the first two stomach emptying samples (i.e., taken after 3.17 and 6.34 min) is negative, as the zeta potential of particles is mainly affected by the pH of the medium [19]. As such, the pH values during the first two stomach emptying times are ca. 8.74 ± 0.19 and 5.39 ± 0.16, which is near the isoelectric point of Lf (at least in the case of the first stomach emptying at 3.17 min into the digestion process). However, pepsin is also present in the medium and its isoelectric point is reported to be ca. 2.7 [20]; some reports indicate that pepsin has an isoelectric point below 1 due to its negative charge at the pH of optimum activity [21]. This means that, at pH values above its isoelectric point, pepsin has a negative charge, which could be the reason for the negative zeta potential of the samples in the first two stomach emptying times. As the pH gets lower, the particles become positively charged since it gets further below the isoelectric point of Lf, which results in a positive zeta potential, and closer to the isoelectric point of pepsin, which increases its zeta potential and becomes less negative. To further analyse the Lf–curcumin nanoparticles’ behaviour during the *in vitro* digestion process, the particle size distribution was analysed, and the results are depicted in Figure 4.

**Figure 5:**
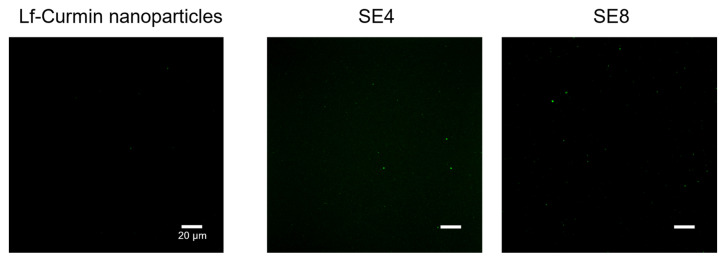
CSLM images of Lf–curcumin nanoparticles during the digestion process. The white bars represent 20 µm.

The analysis of Figure 4 allows for the conclusion that there are significant changes in the particle size distribution when comparing the initial state of Lf–curcumin nanoparticles and the particle distribution throughout the digestion process. In fact, this is in accordance with the results of the particle hydrodynamic size and PdI since, for all stomach emptying times’ samples, there are multiple peaks which may be indicative of particle aggregation [22]. It is also possible to analyse the correlation function (i.e., also called autocorrelation function), since both PdI and particle hydrodynamic measurements are calculated using this model [19], to determine the quality of the DLS results. The analyses of the correlation function of all samples (Figure S1, Supplementary Materials) show that all measurements during the digestion process do not have a good correlation between the calculated and experimental correlation function, which in turn indicates that the DLS results during the digestion process do not present a good quality, i.e., the mathematical model used by the software was not able to predict the hydrodynamic size of the particles with low error. To further assess the Lf–curcumin particles’ aggregation, CSLM was used; the results are depicted in Figure 5, which shows that some degree of particle aggregation occurred during the digestion process since small particles at the micrometre scale became visible.

### 3.3. Curcumin Release during the In Vitro Digestion Process

**Figure 6:**
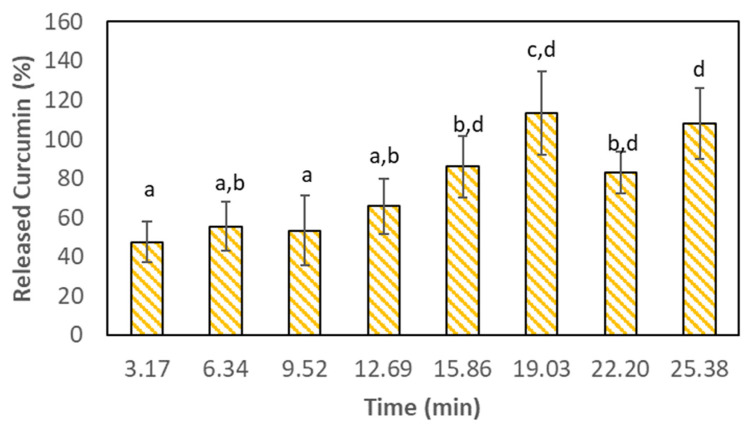
Release of curcumin during the *in vitro* gastric digestion. Columns with the same letters do not present significant statistical differences (*p*-value > 0.05).

In addition to assessing the digestion of Lf, it is important to analyse and quantify the release of curcumin, since one of the most important objectives of a nanodelivery system is protecting the bioactive compounds during the digestion process so that they can be delivered in the intended location (e.g., the small intestine for absorption) [23]. In this regard, curcumin is very well known to have very low bioaccessibility [24] and, as such, its protection from the harsh stomach conditions is crucial to enhance that parameter. Therefore, curcumin was quantified during the gastric digestion process and the results can be seen in Figure 6.

It is possible to observe in Figure 6 that during the first 3.17 min (i.e., the first stomach emptying) of gastric digestion, 47.5 ± 10.5% of curcumin was released. Curcumin was then completely released at 15:52 min of gastric digestion (i.e., the fourth stomach emptying) and no significant statistical differences were found (*p*-value > 0.05) in the subsequent stomach emptying events. This is indicative that, in fact, the Lf–curcumin nanoparticles were destabilized from the first stomach emptying which is in accordance with the DLS results. Furthermore, the Lf–curcumin nanoparticles were further destabilized from minute 15:52, which resulted in the complete release of curcumin. In fact, Lf nanoparticles are often protected in order to resist the gastric digestion conditions and delay the release of its bioactive compound content. For instance, Bourbon et al. [22] studied the addition of a chitosan coating to Lf–glycomacropeptide (Lf-GMP) nanoparticles and used two bioactive compound models, i.e., caffeine and curcumin. The authors observed that only 8.1 ± 0.1% of curcumin was released during *in vitro* gastric digestion. This indicates that chitosan can be used to protect protein nanodelivery systems from the harsh gastric conditions. Similar results were reported by Peng et al. [25], who tested curcumin encapsulated into rice bran albumin and protected with chitosan. The authors concluded that chitosan was able to delay the digestion of the protein nanoparticles in the stomach.

**Figure 7:**
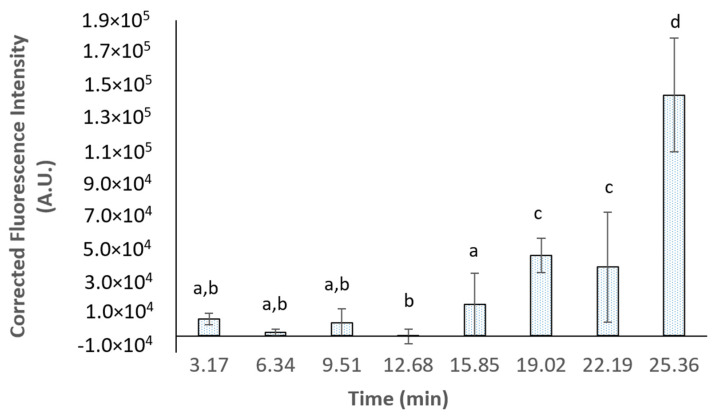
Intrinsic fluorescence of Lf–curcumin nanoparticles during the *in vitro* gastric digestion. Columns with the same letters do not present significant statistical differences (*p*-value > 0.05).

To further investigate the behaviour of Lf–curcumin nanoparticles, the intrinsic fluorescence of Lf–curcumin nanoparticles was measured. The results are depicted in Figure 7 where it is possible to observe that the intrinsic fluorescence of the Lf–curcumin nanoparticles remains constant for the first 15:52 min of gastric *in vitro* digestion. However, at minute 19.02 (i.e., the sixth stomach emptying) there is a significant (*p*-value ≤ 0.05) increase in the fluorescence intensity and a further significant (*p*-value ≤ 0.05) increase at the end of the *in vitro* gastric digestion process. It is also important to notice that the intrinsic fluorescence at minute 12.68 of the *in vitro* gastric digestion is very slightly negative, probably due to a more pronounced influence of pepsin intrinsic fluorescence. The fluorescence curve shape is, in fact, correlated with the curcumin release, at least during the first 19:14 min of the *in vitro* gastric digestion. In fact, the intrinsic fluorescence of Lf–curcumin nanoparticles and the curcumin release until minute 19:14 of the *in vitro* gastric digestion have a high correlation coefficient of 0.871. This indicates that the destabilization of Lf nanoparticles resulted in the complete release of curcumin throughout the *in vitro* gastric digestion. It is also important to mention that both curcumin release and Lf–curcumin nanoparticles’ intrinsic fluorescence have a high standard deviation.

### 3.4. In Situ Assessment of Protein Nanoparticles’ Hydrolysis Using UV-VIS-SWNIR Spectroscopy

The application of fibre optics to evaluate the digestion of food has been shown to be very advantageous due to its rapid and non-destructive nature, without needing the use of sample manipulation [23]. This technique was successfully used here to assess the protein hydrolysis of milk during the *in vitro* gastric digestion process (data not shown due to confidentiality constrains). As such, UV-VIS-SWNIR fibre-optic spectroscopy was used in this study to quantify the protein hydrolysis of Lf–curcumin nanoparticles during the *in vitro* digestion process in real-time. The RFR regression model was used to obtain the estimations of free NH_2_ groups, and the respective results are shown in Table 3.

It is possible to observe from the analysis of Table 3 that the amount of free NH_2_ groups significantly increased (*p*-value ≤ 0.05) from stomach emptying 1 to stomach emptying 2. However, no statistically significant differences (*p*-value > 0.05) were observed when comparing stomach emptying 2 with stomach emptying 3. Although these results are sufficient to accomplish the objective of verifying the adequacy of this measurement technique to assess the protein hydrolysis of Lf–curcumin nanoparticles in real-time, further studies are needed, using higher sample volumes, if the objective is to obtain a more significant picture that represents the hydrolysis of protein nanoparticles during the *in vitro* gastric digestion process.

It is important to mention that some challenges related to the application of this technique in the context of this study were identified, taking into consideration the sample volume used (100 mL); namely, the lack of volume to completely submerge the transmission probe of the fibre optics. Since the RGM was conceptualized to mimic, as closely as possible, the gastric conditions of the human stomach, higher volumes are used during the implementation of the digestion protocol (e.g., 250 mL of sample volume). However, research studies that report the assessment of nanodelivery systems under digestion conditions use low volumes (e.g., 5 mL) [26,27]. In fact, it is important to mention that assessing nanodelivery systems using the RGM can be challenging since this model was idealized to resemble, as closely as possible, the human stomach. As such, high sample volumes are required to implement the *in vitro* digestion protocol in the RGM. This can sometimes be a challenge since some bioactive compounds, especially those who are extracted, are not abundantly available. Furthermore, pure compounds are often used as nanocarriers for bioactive compounds, which can be expensive and, consequently, increase the cost of the digestion analysis when using large volumes. However, other nanosystems can and should be tested in the RGM to further characterize the model and assess its ability to estimate the gastric digestibility of nanodelivery systems. Other applications of this model are also possible, thus significantly increasing its interest as a research tool.

## 4. Conclusions

The *in vitro* RGM was used during this study to assess the gastric digestibility of a nanodelivery system. Lf–curcumin nanoparticles were used to assess the RGM performance. Nanoparticles with a hydrodynamic size of 118.97 ± 12.45 nm, a PdI of 0.24 ± 0.10, and a zeta potential of 10.04 ± 2.16 mV were successfully produced with an association efficiency of 82.10 ± 0.04%. The *in vitro* gastric digestion of this nanodelivery system was subsequently assessed by tracking the particle hydrodynamic size, PdI, zeta potential, intrinsic fluorescence, and curcumin release. Furthermore, an estimation of free NH_2_ groups was conducted using UV-VIS-SWNIR fibre-optic spectroscopy.

The results show that Lf–curcumin nanoparticles were destabilized from the beginning of the digestion process since their particle size and PdI in the first stomach emptying time was 1012.61 ± 858.61 nm and 0.88 ± 0.11, respectively. This was corroborated by the presence of multiple peaks in the Lf–curcumin nanoparticles’ size distribution. Furthermore, negative zeta potential values were observed in the first two stomach emptying time samples, which could be explained by the high negative charge of pepsin, since the gastric pH is far above the isoelectric point of pepsin (i.e., 2.7 or lower) [19,20]. The positive charge of subsequent stomach emptying samples can be attributed to the lower pH values that promote an increase in the surface charge of Lf–curcumin particles (i.e., it gets further away from its isoelectric point), as well as an increase (i.e., becomes less negative) of the surface charge of pepsin. It was also possible to conclude that care must be taken while using DLS during the *in vitro* digestion processes, since the autocorrelation function indicates that the results obtained have a poor quality because the estimated and experimental results are poorly correlated. The CSLM images show that protein aggregation might have happened since small particles became visible at the micrometre scale. The release of curcumin was also quantified and 47.55 ± 10.50% of curcumin was released in the first 3:13 min of digestion with a complete release at 15:52 min. This shows that Lf nanoparticles were not able to protect curcumin during the *in vitro* gastric digestion. In fact, a correlation of 0.871 was found between the release of curcumin and the intrinsic fluorescence of Lf–curcumin nanoparticles, thus showing an increase in the exposure of tryptophan residues. Furthermore, the protein hydrolysis was also measured in real-time, during the first three stomach emptying times. It was possible to observe a significant (*p*-value ≤ 0.05) increase from 0.0681 ± 0.0290 mg·mL^−1^ to 0.1225 ± 0.0002 mg·mL^−1^ of free NH_2_ groups in stomach emptying 1 and stomach emptying 2, respectively. However, there were no further significant (*p*-value > 0.05) differences between stomach emptying 2 and stomach emptying 3.

## Figures and Tables

**Table 1:** Summary of the main *in vitro* digestion conditions.

Fluid	Oral Phase	Gastric Phase	SGF Flow (mL·min^−1^)	Enzymes Flow (mL·min^−1^)	SE Rate (mL·min^−1^)	Number of SE
Electrolyte solution (mL)	6.40 (SSF)	76.40 (SGF)	3.26	0.37	8.09	8
0.3 M CaCl_2_ (µL)	40.00	54.00				
Water (mL)	1.56	18.39				

**Table 2:** Lf–curcumin nanoparticles’ hydrodynamic size, PdI, zeta potential, and association efficiency (*AE*).

Nanoparticle	Hydrodynamic Size (nm)	PdI	Zeta Potential (mV)	*AE* (%)
Lf–Curcumin	118.97 ± 12.45	0.24 ± 0.10	10.04 ± 2.16	82.10 ± 0.04

**Table 3:** Estimated protein hydrolysis values using UV-VIS-SWNIR fibre-optic spectroscopy from stomach emptying 1, 2, and 3 (SE1, SE2, and SE3, respectively).

	SE1	SE2	SE3
Free NH_2_ groups (mg·mL^−1^)	0.0681 ± 0.0290 ^a^	0.1225 ± 0.0002 ^b^	0.2222 ± 0.1389 ^b^

Note: columns with the same superscript letter do not present a statistically significant difference (*p*-value > 0.05).

## Data Availability

The data presented in this study are available on request from the corresponding author. The data are not publicly available due to privacy constrains.

## Supplementary Materials

The following supporting information can be downloaded at: https://www.mdpi.com/article/10.3390/nano13152237/s1, Figure S1: DLS distribution fit for Lf–curcumin samples before (A) and after (B) the digestion process, where the letters from B to I correspond to the stomach emptying from 1 to 8, respectively.
