# Supplementary material for: Assessing the In Vitro Digestion of Lactoferrin-Curcumin Nanoparticles Using the Realistic Gastric Model
Source: Nanomaterials (Basel). 2023 Aug 2;13(15):2237. doi: 10.3390/nano13152237 (PMC10421352; doi:10.3390/nano13152237)
Supplement: Supplementary file 1 [file nanomaterials-13-02237-s001.zip › nanomaterials-2509710-supplementary.pdf]

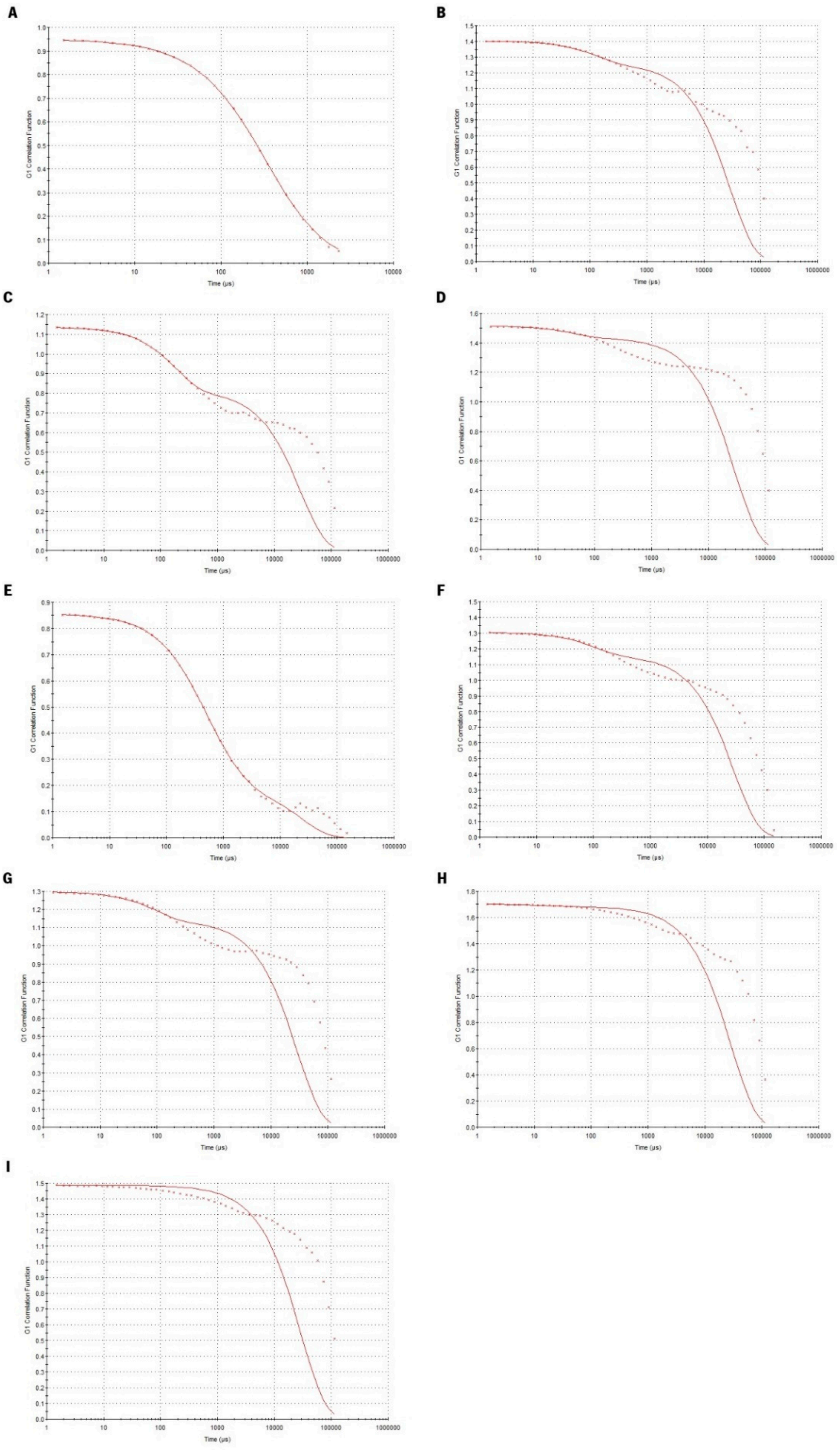

Figure S1 – DLS distribution fit for Lf-curcumin samples before (A) and the digestion process where the letters from B to I correspond to the stomach emptying from 1 to 8, respectively.
